# White matter hyperintensities burden in the frontal regions is positively correlated to the freezing of gait in Parkinson’s disease

**DOI:** 10.3389/fnagi.2023.1156648

**Published:** 2023-04-27

**Authors:** Xiaoya Zou, Zhaoying Dong, Xinwei Chen, Qian Yu, Huimei Yin, Li Yi, Hongzhou Zuo, Jiaman Xu, Xinyi Du, Yu Han, Dezhi Zou, Juan Peng, Oumei Cheng

**Affiliations:** ^1^Department of Neurology, The First Affiliated Hospital of Chongqing Medical University, Chongqing, China; ^2^Department of Radiology, The First Affiliated Hospital of Chongqing Medical University, Chongqing, China

**Keywords:** Parkinson’s disease, freezing of gait, white matter hyperintensities, magnetic resonance imaging, risk factors

## Abstract

**Objective:**

Previous studies have reported that white matter hyperintensities (WMHs) are associated with freezing of gait (FOG), but it is not clear whether their distribution areas have correlations with FOG in Parkinson’s disease (PD) and the potential influencing factors about WMHs.

**Methods:**

Two hundred and forty-six patients with PD who underwent brain MRI were included. Participants were divided into PD with FOG (*n* = 111) and PD without FOG (*n* = 135) groups. Scheltens score was used to assess the WMHs burden in the areas of deep white matter hyperintensities (DWMHs), periventricular hyperintensities (PVHs), basal ganglia hyperintensities (BGHs), and infratentorial foci of hyperintensities (ITF). Whole brain WMHs volume was evaluated by automatic segmentation. Binary logistic regression was used to evaluate relationships between WMHs and FOG. The common cerebrovascular risk factors that may affect WMHs were evaluated by mediation analysis.

**Results:**

There were no statistical differences between PD with and without FOG groups in whole brain WMHs volume, total Scheltens score, BGHs, and ITF. Binary logistic regression showed that the total scores of DWMHs (OR = 1.094; 95% CI, 1.001, 1.195; *p* = 0.047), sum scores of PVHs and DWMHs (OR = 1.080; 95% CI, 1.003, 1.164; *p* = 0.042), especially the DWMHs in frontal (OR = 1.263; 95% CI, 1.060, 1.505 *p* = 0.009), and PVHs in frontal caps (OR = 2.699; 95% CI, 1.337, 5.450; *p* = 0.006) were associated with FOG. Age, hypertension, and serum alkaline phosphatase (ALP) are positively correlated with scores of DWMHs in frontal and PVHs in frontal caps.

**Conclusion:**

These results indicate that WMHs distribution areas especially in the frontal of DWMHs and PVHs play a role in PD patients with FOG.

## 1. Introduction

Parkinson’s disease (PD) is a common neurodegenerative disease in the elderly. Freezing of gait (FOG) is one of the most disabling motor symptoms in PD, which often occurs in the advanced stage (Bloem et al., 2004; Perez-Lloret et al., 2014). Its main feature is the difficulty of autonomous gait leading to sudden, short, and accidental interruption of walking, which often occurs at the beginning of gait or turning (Okuma et al., 2018; Kim et al., 2019). It significantly reduces the quality of life in patients with PD (Rahman et al., 2008; Perez-Lloret et al., 2014). Previous studies have shown that male (Macht et al., 2007), low education level (Zhang et al., 2016), longer disease duration (Contreras and Grandas, 2012; Perez-Lloret et al., 2014), movement severity (Perez-Lloret et al., 2014), cognitive decline (Qu et al., 2022), depression (Zhang et al., 2016), and anxiety (Ehgoetz Martens et al., 2018) may increase the risk of FOG in PD patients (Zhang et al., 2016). Despite previous research, inconsistencies have been identified in the existing findings on FOG (Ehgoetz Martens et al., 2018; Ou et al., 2018; Herman et al., 2019). Meanwhile, the therapeutic efficacy of FOG remains unsatisfactory. Therefore, it is necessary to explore the relevant factors of FOG and provide references for treatments of FOG.

Recently, the role of white matter hyperintensities (WMHs) in PD symptoms has been gradually discovered. WMHs are usually detected on magnetic resonance imaging (MRI) of the brain in the elderly (Abraham et al., 2016; Raman et al., 2016). The underlying pathology of WMHs is mainly attributed to axonal loss and demyelination, resulting from chronic ischemia of cerebral small vessel diseases (CSVD; de Schipper et al., 2019; Lee et al., 2020). In addition, it is hypothesized that the interplay between blood–brain barrier dysfunction, inflammation, amyloid angiopathy, inheritance, as well as liver and kidney functions, may also contribute to the underlying causes of WMHs (Sala et al., 2016; Abdeldyem et al., 2017; Wardlaw et al., 2019; Zhang et al., 2022). This implies that external interventions may offer viable strategies to prevent and treat WMHs. These observations have stimulated our interest in exploring of WMHs. As the most common neuroimaging biomarker of CSVD, WMHs have been reported to be associated with cognitive decline and severe motor symptoms in PD patients (Malek et al., 2016; Hou et al., 2022). Moreover, patients with a significant burden of WMHs have been reported to have elevated scores of axial symptoms and bradykinesia (Bohnen et al., 2011; Arena et al., 2016; Moccia et al., 2016; Lee et al., 2020). Recent investigations have also established a strong association between WMHs and gait disorder in patients with (Pozorski et al., 2019; Dadar et al., 2020; Jeong et al., 2021) or without PD (Baezner et al., 2008; Willey et al., 2013). Simultaneously, the location of WMHs has also been implicated in gait disorder. Kim et al. found that the score of periventricular hyperintensities (PVHs) is positively correlated with gait disorder in PD (Kim et al., 2016).

In a retrospective study including 423 *de novo* PD patients from the Parkinson’s Progression Markers Initiative (PPMI), it was revealed that approximately 41.84% of the patients developed FOG during the four-year follow-up period. Moreover, the PD patients with FOG had a higher baseline WMHs burden than those without FOG (PD-nFOG; Dadar et al., 2021). In a prospective study involving 268 *de novo* PD patients, it was shown that the proportion of subsequent FOG reached 35.71% in the group with severe WMHs at baseline, after a follow-up period of 3 years. Conversely, the group with mild WMHs accounted for only 13.64% of subsequent FOG (Chung et al., 2019). However, there is still a lack of cross-sectional studies. In this regard, our study aims to evaluate whether the WMHs burden is more serious in PD-FOG than in PD-nFOG. We also seek to determine whether the regions of WMHs have impacts on PD-FOG. In addition, we analyzed which common cerebrovascular disease risk factors are related to WMHs in PD patients and explored mediated factors that impact FOG through WMHs.

## 2. Methods

### 2.1. Participants

We recruited PD patients between June 2020 and March 2022 from the First Affiliated Hospital of Chongqing Medical University. All eligible patients had undergone brain MRI at this hospital and completed Movement Disorder Specialist-Unified Parkinson’s Disease Rating Scale (MDS-UPDRS), as well as screening for cerebrovascular risk factors at the same time. All patients in this study were assessed for MDS-UPDRS during the OFF stage.

PD was diagnosed according to the clinical diagnostic criteria of Movement Disorder Specialist in 2015 by an experienced neurologist. Exclusion criteria included previous cerebral infarction, cerebral hemorrhage, brain trauma, hydrocephalus, intracranial space-occupying, mental diseases, dementia, other neurological or musculoskeletal diseases that may affect gait, inability to walk alone, and the laboratory indexes of liver and kidney 3 times the normal level.

The presence of motor symptoms by means of the Hoehn & Yahr (H&Y) scale and the MDS-UPDRS part III (Liepelt-Scarfone et al., 2015) were assessed by neurologists with expertise in movement disorders. Four main types of motor impairment (involving tremor, rigidity, bradykinesia, and axial symptoms) were classified according to MDS-UPDRS part III (Stebbins et al., 2013). Cognitive impairment, depression, anxiety, and Rapid-eye-movement (REM) sleep behavior disorder were, respectively, evaluated by means of the Mini-Mental State Examination (MMSE; Folstein et al., 1975; Arevalo-Rodriguez et al., 2021), Hamilton Depression Scale (HAMD; Hamilton, 1976) and Hamilton Anxiety Scale (HAMA; Hamilton, 1959), and Rapid-eye-movement Sleep Behavior Disorder Screening Questionnaire (RBDSQ; Stiasny-Kolster et al., 2007). The L-dopa equivalent daily dose (L-dopa) in each patient was calculated according to standardized procedures (Tomlinson et al., 2010). This study was approved by the Ethics Committee of the First Affiliated Hospital, Chongqing Medical University, China, in accordance with the Declaration of Helsinki. Written informed consent was obtained from all the participants.

### 2.2. Freezing of gait

In PD patients, freezing of gait (FOG) was diagnosed based on clinical examination and a nonzero score on item 3.11 “Freezing of Gait” of the MDS-UPDRS Part III in the OFF stage. The severity of FOG was assessed by means of the FOG Questionnaire (FOG-Q; Giladi et al., 2000).

### 2.3. White matter hyperintensities burden

The brain MRI, including FLAIR sequence, was on the 1.5 T (Avanto, Siemens AG) or 3.0 T scanner (Verio, Siemens AG). The Scheltens scale scores MRI hyperintensities according to size and number of lesions in four locations: PVHs, DWMHs, basal ganglia hyperintensities (BGHs), and infratentorial foci of hyperintensities (ITF; Scheltens et al., 1993). DWMH (score: 0–24) is divided into the frontal (DWMHs-frontal), parietal (DWMHs-parietal), temporal (DWMHs-temporal), and occipital lobes (DWMHs-occipital), while the score of each lobe is 0–6 (score 0 = no abnormality; 1 = lesion <4 mm, ≤ 5 lesions; 2 = lesion <4 mm, > 5 lesions; 3 = 4 mm ≤ lesion ≤10 mm, ≤ 5 lesions; 4 = 4 mm ≤ lesion ≤10 mm, > 5 lesions; 5 = lesion >10 mm, ≥ 1 lesion; 6 = fusion lesion). PVHs (score: 0–6) were scored in the anterior (PVHs-frontal caps), posterior (PVHs-occipital caps) horns of the lateral ventricles, and along the lateral ventricles (PVHs-lateral ventricles bands), while a score of each part is 0–2 (0 = none; 1 = lesion ≤5 mm; 2 = 5 mm < lesion <10 mm). The rating criteria of DWMHs were also applied to regions of the BGHs (caudate, putamen, globus pallidus, thalamus, and internal capsule) and ITF (cerebellum, midbrain, pons, and medulla). To evaluate the impact of PVHs and DWMHs, the sum of PVHs and DWMHs scores (P&D WMHs score) is calculated in this study. The WMHs rating was performed by two imaging physicians who were unknown about the clinical information. If the score was discordant between the raters, the final score was determined by consensus.

Whole brain white matter hyperintensity volume (WMHV) at baseline was also used as a marker of WM pathology. Lesions were segmented by the lesion prediction algorithm [Chapter 6.1 (Schmidt, 2017)] as implemented in the LST toolbox version 3.0.0^1^ for SPM. This algorithm consists of a binary classifier in the form of a logistic regression model trained on the data of 53 multiple sclerosis patients with severe lesion patterns. Data were obtained at the Department of Neurology, Technische Universität München, Munich, Germany. As covariates for this model, a similar lesion belief map as for the lesion growth algorithm was used as well as a spatial covariate that takes into account voxel specific changes in lesion probability (Schmidt et al., 2012). Parameters of this model fit are used to segment lesions in new images by providing an estimate for the lesion probability for each voxel.

### 2.4. Cerebrovascular risk factors

Common risk factors that have been previously reported were selected as risk factors for this study including the history of smoking, hypertension, diabetes, heart disease, and whether to use lipid-lowering drugs (Wardlaw et al., 2014, 2019). The biochemical indexes contained body mass index (BMI), blood lipid indicators, high-sensitivity c-reactive protein (hsCRP), and relevant liver and kidney function indicators (Sala et al., 2016; Abdeldyem et al., 2017; Wardlaw et al., 2019; Zhang et al., 2022).

### 2.5. Statistical analyses

Statistical analyses were performed using SPSS 25.0 software. Data were recorded as mean ± standard deviation or median (interquartile range). Chi-square test was used for baseline demographics and other counting data. Independent t-tests and Mann–Whitney *U*-test were used to analyze differences between groups. *p* < 0.05 was considered statistically significant. The associations between WMHs and FOG were assessed using binary logistic regression after adjusting for disease duration, sex, and other influencing factors. All indicators were tested for collinearity. Because of the multicollinearity of the two WMHs scores, each regression model had one of the WMHs scores as the independent variable. Other statistically significant values (*p* < 0.05) in univariate analysis were included in binary logistic regression as confounding variables, whereas with or without FOG in PD was set as a dependent variable (PD-FOG = 1, PD-nFOG = 0).

The associations between WMHs and risk factors were assessed using Spearman’s correlation analysis. Multivariable linear regression analysis was used to evaluate which WMHs indicators were correlated with the FOG-Q in PD-FOG group. Ordinal logistic regression and multivariable linear regression were used to analyze the relationships between cerebrovascular risk factors and WMHs according to the type of dependent variable. *p* < 0.05 was statistically significant. According to the correlations and the regression models, we used mediation analysis to test the hypothesis that the cerebrovascular risk factors impact FOG through WMHs.

## 3. Results

### 3.1. Demographic, clinical characteristics, and cerebrovascular risk factors data

Two hundred and forty-six participants were included in this study. The clinical and demographic characteristics of the subjects are presented in Table 1. Among the participants, 111 patients were PD-FOG (43 males), and 135 patients were PD-nFOG (75 males). Compared with PD-nFOG, PD-FOG patients had longer disease duration, a higher proportion of women, higher H&Y grade, higher levodopa dosage, more severe bradykinesia and axial symptoms, higher scores of HAMD, HAMA, and FOG-Q. For cerebrovascular risk factors in Table 2, the Serum alkaline phosphatase (ALP), lactate dehydrogenase (LDH), apolipoprotein A-1 (ApoA1), and lipoprotein-α [Lp (α)] values were higher in PD-FOG, but the uric acid (UA) level was lower.

**Table 1:** Comparison of demographic and clinical characteristics of the two participant groups.

Variables	PD(*n* = 246)	PD-nFOG(*n* = 135)	PD-FOG(*n* = 111)	*p*
Sex (male/female)	118/128	75/60	43/68	0.009^**^
Age, year	66.26 ± 8.62	66.61 ± 8.61	65.82 ± 8.64	0.637
**Degree of education**				
Illiterate	20	11	9	0.863
Primary	47	26	21	
Junior	78	39	39	
Senior	64	37	27	
University	37	22	15	
Disease duration, year	3(1.88,6)	2(1,4)	5(2,9)	<0.001^**^
H&Y	2(1.5,2.5)	2(1,2)	2.5(2,3)	<0.001^**^
L-dopa	312.50(150,550)	300(0,450)	450(300,650)	<0.001^**^
**MDS UPDRS score**				
MDS-UPDRS Part I	8(5,13)	7(4,11)	10(6,15)	<0.001^**^
MDS-UPDRS Part II	11(7.17)	9(6,14)	15(10,21)	<0.001^**^
MDS-UPDRS Part III	20(11.75,32.25)	17(9,30)	26(16,43)	<0.001^**^
**Motor subtype**				
UPDRS tremor	2(0,4)	2(0,4)	1(0,4)	0.164
UPDRS rigidity	3(1,6)	3(0,6)	3(1,7)	0.244
UPDRS bradykinesia	12(4.75,25)	8(3,17)	22(9,44)	<0.001^**^
UPDRS axial	2(1,4)	1(0,2)	4(2,6)	<0.001^**^
MMSE	27(24,29)	28(24,29)	27(23,29)	0.113
HAMD	5(2,9)	4(2,8)	6(2,12)	0.03^*^
HAMA	4(1,9)	4(1,7)	5(2,12)	0.014^*^
RBDSQ	2(1,6)	3(1,5)	2(1,6)	0.341
FOG-Q	3(1,10)	2(1,3)	10(5,16)	<0.001^**^

Data are numbers (*N*), mean ± standard deviation or median (interquartile range). Analyzed by Chi-square test, independent *t*-tests and Mann–Whitney *U-*test; PD-FOG, PD patients with freezing of gait; PD-nFOG, PD patients without freezing of gait; H&Y, Hoehn and Yahr scale; L-dopa, The L-dopa equivalent daily dose; MDS-UPDRS, Unified Parkinson’s disease rating scale; MMSE, mini-mental state examination; HAMD, Hamilton depression scale; HAMA, Hamilton Anxiety Scale; RBDSQ, Rapid-eye-movement Sleep Behavior Disorder Screening Questionnaire; FOG-Q, Freezing of Gait Questionnaire; ^**^p < 0.01, ^*^p < 0.05.

**Table 2:** Comparison of cerebrovascular risk factors of the two participants groups.

Variables	PD(*n* = 246)	PD-nFOG(*n* = 135)	PD-FOG(*n* = 111)	*p*
**History**				
Hypertension (yes/no)	212/34	119/16	93/18	0.324
Diabetes (yes/no)	227/19	121/14	106/5	0.086
Family history of cardiovascular and cerebrovascular diseases (yes/no)	203/43	106/29	97/14	0.068
Smoke (yes/no)	172/74	96/39	76/35	0.653
Lipid-lowering drugs (yes/no)	159/87	90/45	69/42	0.462
**Blood fat**				
TC, mmol/L	4.26(3.66,4.95)	4.24(3.61,4.94)	4.26(3.69,5.04)	0.471
TG, mmol/L	0.96(0.75,1.31)	0.94(0.72,1.24)	0.98(0.79,1.34)	0.144
HDL-C, mmol/L	1.42(1.17,1.74)	1.41(1.16,1.71)	1.44(1.19,1.80)	0.493
LDL-C, mmol/L	2.56(1.99,3.58)	2.56(1.98,3.06)	2.51(2.02,3.13)	0.793
ApoA1, g/L	1.44(1.26,1.62)	1.41(1.24,1.58)	1.50(1.3,1.65)	0.030^*^
Apo-B, g/L	0.82(0.65,0.99)	0.79(0.63,0.96)	0.82(0.66,1.02)	0.207
Lp(α), mg/L	69.50(33.75,182.00)	62(32,149)	98(39,257)	0.031^*^
**Liver**				
Alb, g/L	43.00(40.00,45.00)	43(40,45)	44(42,46)	0.483
ALT, U/L	15.00(11.00,21.00)	16(12,24)	15(11,20)	0.072
AST, U/L	19.00(16.00,22.00)	19(16,22)	19(16,23)	0.956
ALP, U/L	66.00(56.00,79.00)	65(55,76)	69(59,89)	0.01^*^
LDH, U/L	164.00(148.00,194.00)	160(145,182)	170(152,205)	0.001^**^
**Kidney**				
Urea, mmol/L	5.70(4.80,6.60)	5.5(4.5,6.6)	5.9(4.9,6.6)	0.090
Crea, μmol/L	66.00(57.00,76.00)	66(59,77)	66(56,74)	0.151
UA, μmol/L	280.50(233.75,336.00)	290(246,347)	268(228,328)	0.025^*^
Cys-C, mg/L	0.82(0.73,0.92)	0.28(0.73,0.93)	0.82(0.74,0.92)	0.942
eGFR, mL/(min × 1.73 m2)	94.65(84.95,101.80)	95.5(85.7,102.3)	92.6(82.6,101.6)	0.497
hsCRP, mg/L	0.54(0.31,1.11)	0.49(0.31,0.91)	0.58(0.3,2.01)	0.089
BMI, kg/m^2^	22.50(20.41,24.44)	22.31(20.45,24.03)	23.15(20.31,24.65)	0.238

Data are numbers (*N*) or median (interquartile range). TC, total cholesterol; TG: triglycerides; HDL-C, high-density lipoprotein; LDL-C, low-density lipoprotein; Apo-B, Apolipoprotein B; ApoA1, Apolipoprotein A-1; Lp (α), Lipoprotein-α; Alb, albumin; ALT, alanine aminotransferase; AST, Aspartate aminotransferase; ALP, Serum alkaline phosphatase; LDH, Lactate dehydrogenase; UA, uric acid; Crea, creatinine; Cys-C, Cystatin C; eGFR, estimated Glomerular Filtration Rate; hsCRP, high-sensitivity c-reactive protein; BMI, Body Mass Index; ^**^p < 0.01, ^*^p < 0.05.

### 3.2. Relationships between WMHs and FOG

Total Scheltens score and P&D WMHs score were higher in PD-FOG. Additionally, PVHs-frontal caps, DWMHs, and DWMHs-frontal scores were found to be significantly higher in PD-FOG as compared to PD-nFOG (Table 3). Statistically significant variables in Table 1 such as gender were adjusted. Five regression models were developed, and the results are presented in Supplementary Table 1. The results showed that the scores of P&D WMHs (OR = 1.080; 95% CI, 1.003 to 1.164; *p* = 0.042), DWMHs (OR = 1.094; 95% CI, 1.001 to 1.195; *p* = 0.047), DWMHs-frontal (OR = 1.263; 95% CI, 1.060 to 1.505 *p* = 0.009), and PVHs-frontal caps (OR = 2.699; 95% CI, 1.337 to 5.450; *p* = 0.006) were the independent risk factors for PD-FOG (Supplementary Table 1). But the total Scheltens score was not associated with FOG (OR = 1.067; 95% CI, 0.994 to 1.144; *p* = 0.071).

**Table 3:** Comparison of whole brain white matter hyperintensity volume and WMHs scores of the two participants groups.

Variables	PD(*n* = 246)	PD-nFOG(*n* = 135)	PD-FOG(*n* = 111)	*p*
Whole brain WMHV, mL	3.06(1.35,7.65)	2.8(1.45,7.45)	3.52(1.17,7.65)	0.831
Total Scheltens score	8.47 ± 5.30	7.66 ± 5.12	9.45 ± 5.37	0.006^**^
P&D WMHs	8.05 ± 4.95	7.27 ± 4.79	8.99 ± 5.00	0.006^**^
PVHs	3.78 ± 1.31	3.63 ± 1.36	3.97 ± 1.22	0.084
Frontal caps	1.31 ± 0.54	1.18 ± 0.53	1.47 ± 0.52	<0.001^**^
Occipital caps	1.11 ± 0.67	1.1 ± 0.71	1.12 ± 0.63	0.941
Lateral ventricles bands	1.37 ± 0.59	1.35 ± 0.62	1.39 ± 0.56	0.725
DWMHs	4.26 ± 4.19	3.64 ± 4.04	5.02 ± 4.27	0.010^*^
Frontal	2.27 ± 2.18	1.79 ± 1.96	2.86 ± 2.30	<0.001^**^
Parietal	1.48 ± 2.04	1.38 ± 2.03	1.61 ± 2.04	0.405
Temporal	0.13 ± 0.57	0.1 ± 0.42	0.18 ± 0.70	0.473
OcelpLtal	0.37 ± 1.15	0.38 ± 1.09	0.37 ± 1.22	0.384
BGHs	0.27 ± 0.75	0.21 ± 0.77	0.35 ± 0.72	0.054
Caudate	0.00 ± 0.00	0.00 ± 0.00	0.00 ± 0.00	-
Putamen	0.06 ± 0.33	0.01 ± 0.12	0.11 ± 0.47	0.178
Globus	0.05 ± 0.27	0.04 ± 0.28	0.06 ± 0.24	0.091
Pallidus	0.11 ± 0.40	0.10 ± 0.32	0.14 ± 0.48	0.353
Thalamus	0.05 ± 0.40	0.59 ± 0.45	0.05 ± 0.31	0.554
ITF	0.15 ± 0.46	0.18 ± 0.47	0.11 ± 0.43	0.178
Infratentorial regions	0.11 ± 0.38	0.13 ± 0.35	0.10 ± 0.40	0.295
Midbrain	0.00 ± 0.00	0.00 ± 0.00	0.00 ± 0.00	-
Pons	0.03 ± 0.18	0.05 ± 0.22	0.01 ± 0.09	0.076
Medulla	0.00 ± 0.00	0.00 ± 0.00	0.00 ± 0.00	-

Data are mean ± standard deviation or median (interquartile range). WMHV, white matter hyperintensity volume; WMHs, white matter hyperintensities; PVHs, periventricular hyperintensities; DWMHs, deep white matter hyperintensities; BGHs, basal ganglia hyperintensities; ITF, infratentorial foci of hyperintensities; P&D WMHs, the scores of PVHs and DWMHs; ^**^p < 0.01, ^*^p < 0.05; “-,” The mean value is zero and cannot be calculated.

In the PD-FOG group, Spearman’s correlation analysis showed that FOG-Q was related to the whole brain WMHV (r = 0.213, *p* = 0.025), but further regression showed that WMHV was not independently associated with FOG-Q in multivariable linear regression models. Furthermore, FOG-Q did not show a significant difference in DWMHs-frontal (r = 0.076, *p* = 0.429) and PVHs-frontal cap (r = 0.134, *p* = 0.162).

### 3.3. Relationships between WMHs, clinical factors, and risk factors in PD

In Spearman’s correlation analysis, regarding the clinical indicator (Table 4), age was correlated with PVHs-frontal caps, DWMHs-frontal, P&D WMHs scores, and total Scheltens score. L-dopa equivalent dose was interrelated with the DWMHs-frontal score. H&Y was positively interrelated with PVHs-frontal caps, P&D WMHs score, and total Scheltens score. In motor sub scores of MDS-UPDRS, DWMHs-frontal and PVHs-frontal caps scores were correlated with bradykinesia (r = 0.171, *p* = 0.007; r = 0.13, *p* = 0.042) and axial symptom (r = 0.22, *p* < 0.001; r = 0.197, *p* = 0.002; Supplementary Figure 1).

**Table 4:** Correlation analysis about the risk factors of WMHs in PD.

Related variables	PVHs-frontal caps		DWMHs-frontal		P&D WMHs scores		Total Scheltens score	
	r value	*p*-value	r value	*p*-value	r value	*p*-value	r value	*p*-value
Sex	0.012	0.854	0.072	0.260	−0.046	0.470	−0.0.059	0.355
Age	0.230	<0.001^**^	0.348	<0.001^**^	0.457	<0.001^**^	0.464	<0.001^**^
Disease duration	0.100	0.117	0.071	0.269	0.107	0.093	0.120	0.061
L-dopa	0.079	0.217	0.134	0.035^*^	0.102	0.111	0.106	0.096
H&Y	0.177	0.005^**^	0.115	0.073	0.131	0.041^*^	0.142	0.026^**^
Hypertension	0.215	0.001^**^	0.133	0.037^*^	0.209	0.001^**^	0.204	0.001^**^
Diabetes	0.108	0.092	0.082	0.199	0.095	0.138	0.099	0.120
Smoke	0.009	0.886	−0.031	0.634	0.008	0.896	0.012	0.851
Family history of cardiovascular and cerebrovascular diseases	0.065	0.308	0.062	0.334	0.049	0.449	0.062	0.329
Lipid-lowering drugs	0.154	0.016^*^	0.087	0.174	0.108	0.090	0.118	0.066
**Liver**								
Alb	0.064	0.316	−0.046	0.468	−0.073	0.253	−0.071	0.269
AST	0.078	0.220	0.130	0.041^*^	0.163	0.011^*^	0.176	0.006^**^
ALP	0.134	0.036^*^	0.196	0.002^**^	0.163	0.011^*^	0.170	0.008^**^
LDH	0.110	0.086	0.238	<0.001^**^	0.225	<0.001^**^	0.227	<0.001^**^
**Kidney**								
Crea	0.150	0.018^*^	0.041	0.524	0.156	0.014^*^	0.172	0.007^**^
Cys-C	0.101	0.113	0.090	0.161	0.148	0.020^*^	0.161	0.012^*^
eGFR	−0.214	0.001^**^	−0.179	0.005^**^	−0.264	<0.001^**^	−0.278	<0.001^**^
hsCRP	0.120	0.061	0.179	0.005^**^	0.197	0.002^**^	0.215	0.001^**^

There is no significant correlation between the five indicators of WMHs, include PVHs-frontal caps, DWMHs-frontal, DWMHs, P&D WMHs scores, and total Scheltens score and risk factors, which included sex, disease duration, smoking, family history of cardiovascular, cerebrovascular diseases and other risk factors not shown in this table. ^**^p < 0.01, ^*^p < 0.0.

**Figure 1:**
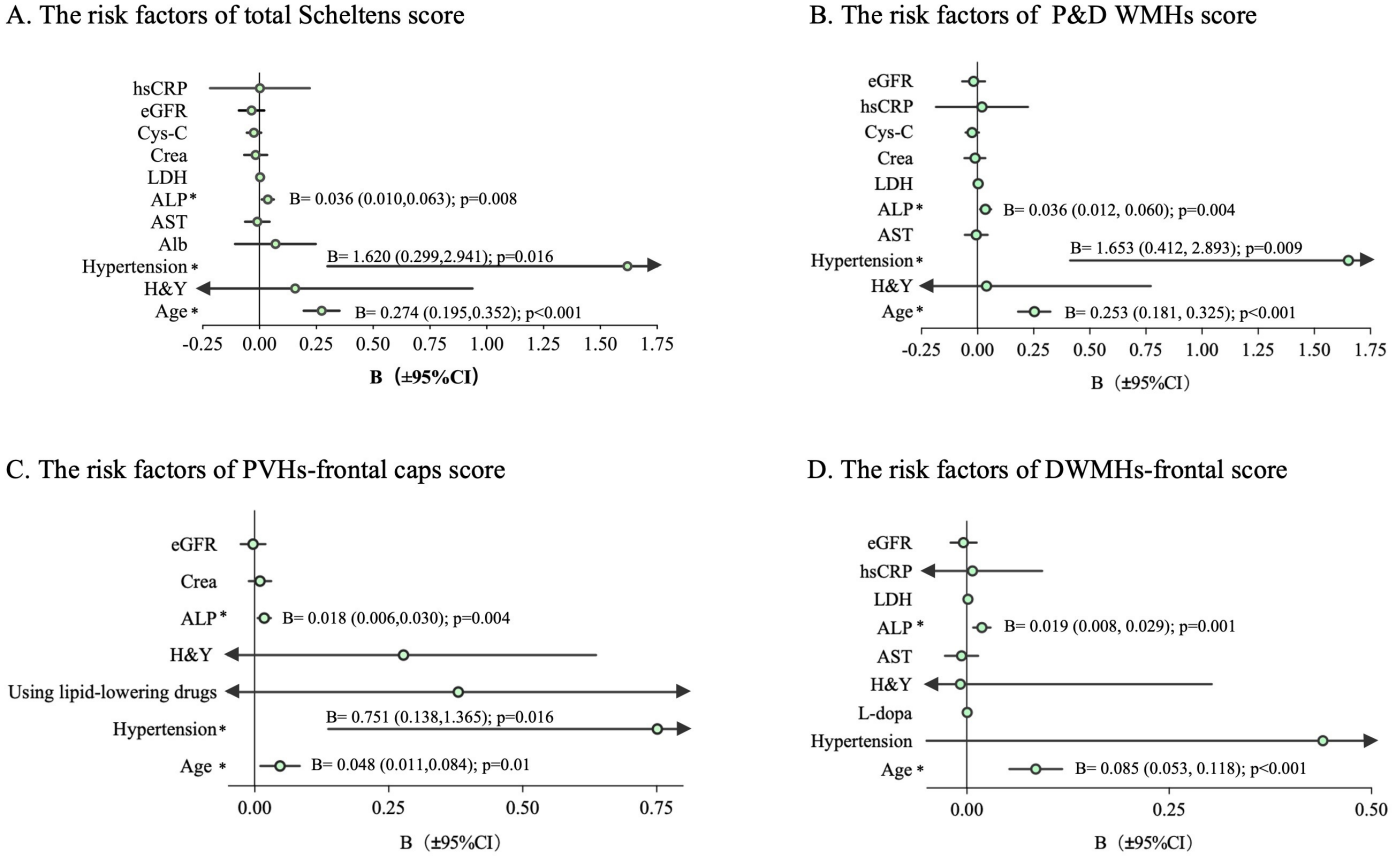
Regression analysis to determine the risk factors of each WMHs score in PD. The bar represents a 95% CI of B. The arrow indicates the value beyond the x-axis area. CI, confidence interval; *p < 0.05; **(A)** The risk factors of total Scheltens score; **(B)** The risk factors of P&D WMHs score; **(C)** The risk factors of PVHs-frontal caps score; **(D)** The risk factors of DWMHs-frontal score; B, regression coefficient.

The correlation between cerebrovascular risk factors and the five WMHs indicators is presented in Table 4. Cerebrovascular risk factors that may lead to WMHs in Table 4 were included in the regression model, as well as age, H&Y, and L-dopa equivalent dose. The total Scheltens score (Figure 1A) as well as P&D WMHs score (Figure 1B) were found to be associated with age, hypertension, and ALP. Age (B = 0.048; 95% CI, 0.011 to 0.084, *p* = 0.01), hypertension (B = 0.751; 95% CI, 0.138 to 1.365, *p* = 0.016), and ALP (B = 0.018; 95% CI, 0.006 to 0.030, *p* = 0.004) were related to PVH-frontal caps (Figure 1C). Age (B = 0.085; 95% CI, 0.053 to 0.118, *p* < 0.001) and ALP (B = 0.019; 95% CI, 0.008 to 0.029, *p* = 0.001) increased the risk of DWMHs-frontal score (Figure 1D). Meanwhile, age and eGFR were associated with whole brain WMHV (Supplementary Figure 2).

### 3.4. Relationships between WMHs, cerebrovascular risk factors, and FOG

**Figure 2:**
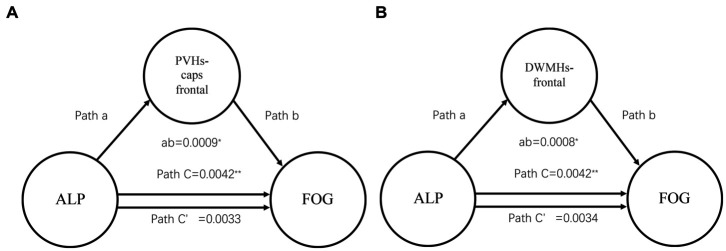
Mediation analysis about WMHs scores and ALP showing the mediation effects of ALP on FOG through WMHs. WMHs, white matter hyperintensities; ALP, Serum alkaline phosphatase; PVHs, periventricular hyperintensities; DWMHs, deep white matter hyperintensities; ^**^p < 0.01, ^*^p < 0.05; **(A)** Mediation analysis about PVHs-frontal caps score and ALP; **(B)** Mediation analysis about DWMHs-frontal score and ALP.

The mediation analysis displayed the effect of ALP on FOG via PVHs-frontal caps and DWMHs-frontal scores. In Figure 2A, the average causal mediation effect of PVHs-frontal caps was obtained: ab = 0.0009, 95% CI: 0.0001, 0.0017, *p* = 0.025. The intermediary effect accounted for 21.43% of the total effect (Figure 2A). About DWMHs-frontal, the average causal mediation effect was that ab = 0.0008, 95% CI: 0.00007, 0.0015, *p* = 0.033. The intermediary effect accounted for 19.05% of the total effect (Figure 2B). Meanwhile, there was no significant effect of hypertension on FOG via PVHs-frontal caps and DWMHs-frontal scores.

## 4. Discussion

In this study, we explored the relationships between the regions of WMHs and FOG in PD patients. Our findings indicate that PD-FOG patients exhibit a more severe burden of P&D WMHs than PD-nFOG. Specifically, PD-FOG manifested a greater burden of DWMHs and PVHs in frontal regions, compared to PD-nFOG. It was suggested that the WMHs in frontal regions may be involved in the pathological mechanism of FOG. Furthermore, hypertension, age, and ALP were correlated with WMHs. These findings may inform the direction of intervention and treatment strategies for FOG.

At present, the mechanism about the development of FOG in PD remains ambiguous (Jacobs et al., 2009; Lewis and Barker, 2009; Plotnik et al., 2012; Nieuwboer and Giladi, 2013; Vandenbossche et al., 2013). Previous studies have found that the WMHs at the baseline are related to the future FOG in PD (Chung et al., 2019; Dadar et al., 2021). At the same time, our cross-sectional study showed that there was a statistical difference between PD-FOG and PD-nFOG with regard to gender, disease duration, H&Y, L-dopa, MDS UPDRS scores, etc. WMHs were still related to FOG after adjustment for these factors. The major supraspinal regions playing key roles in locomotion are the mesencephalic locomotor region (MLR), pontomedullary reticular formation (PMRF), basal ganglia, cerebellum, and cerebral cortex (Nutt et al., 2011). The supplementary motor area (SMA) and premotor area (PM) are associated with movement initiation (Brooks and Stoney, 1971). Regarding BGHs and ITF, our research did not find a difference between PD-FOG and PD-nFOG. A large cohort study also found that DWMHs-frontal and PVHs, but not BGHs and ITF, were associated with a history of falls in nondisabled elderly (Blahak et al., 2009). It may be due to white matter changes that destroy the cerebral cortex and interrupt the connection between primary motor cortex and SMA to basal ganglia and cerebellum (Thompson and Marsden, 1987).

We also found that the scores of DWMHs-frontal and PVHs-frontal caps were bonded to PD-FOG, but not with the entire brain WMHs. In addition, the generation of motor programs by PM and SMA is contingent upon the timely and accurate integration of vision, proprioceptive, and vestibular sensations into the temporoparietal cortex (Takakusaki, 2013). A DTI study revealed the existence of white matter damage existed in the majority of frontoparietal and temporo-occipital cortico-cortical connections in PD-FOG as compared to PD-nFOG (Canu et al., 2015), while DWMHs-frontal potentially causing injury to those junctions. The indirect effects of periventricular and frontal WMHs on bradykinesia may be explained by changes in cortical or striatal activity (Cacciola et al., 2016; Foffani and Obeso, 2018). With respect to cortical activity, the explosive activity induced by glutamatergic neurons from the medial prefrontal cortex promotes the phasic dopamine release from the inferior substantia nigra compacta, which is important for the control of random motor performance. A recent study found that WMHs may not only contribute to the occurrence and development of motor symptoms by reducing the level of dopaminergic neurons in the substantia nigra of PD, but also may participate in the pathophysiological mechanism of non-dopaminergic neurons. Furthermore, the frontal and periventricular WMHs were associated with dopamine transporter availability (DAT) in striata subregions (Jeong et al., 2021). Hence, it is plausible that the presence of WMHs in the frontal regions may exert both direct and indirect impact through DAT, thus causing the appearance of FOG. Furthermore, it has been postulated that PVHs in the frontal may influence gait in PD, a hypothesis that is supported by the observation of similar gait abnormalities in other disorders, such as normal pressure hydrocephalus (NPH), which primarily involves periventricular areas. Based on the anatomical structure of the periventricular area, compression of descending periventricular fibers from the frontal motor area or dysfunction of the ascending basal ganglia ring may be the cause of gait disorder in NPH (Alexander et al., 1986; Curran and Lang, 1994; Stolze, 2001). Meanwhile, the medical treatment that reverses PVHs volume improves gait in patients with NPH (Gilbert et al., 2014).

Our investigation revealed that several cerebrovascular risk factors, including age, hypertension, ALP, and eGFR, were related to WMHs, which were consistent with previous studies (Zhuang et al., 2018). Additionally, our study found that eGFR was linked to WMHV. However, our analysis did not identify age, hypertension, eGFR, diabetes, or smock as significant factors in relation to PD-FOG, except for ALP. Mediation analysis prompted that ALP influences FOG through WMHs in PVHs-frontal caps and DWMHs-frontal. ALP inactivates organic pyrophosphate, which is an important inhibitor of vascular calcification (Lomashvili et al., 2008; Shimizu et al., 2013). Vascular medial calcification promotes arteriosclerosis which may lead to the occurrence of various cerebral ischemia, including the WMHs (Ohmine et al., 2008). Previous studies have shown that higher serum levels of ALP are more likely to have a large WMH volume (Ryu et al., 2014) and thus leading to a high risk of silent lacunar infarct and WMHs (Xiangyu et al., 2019). In addition, a recent study suggested that the relationships of ALP and CRP were independent of each other in cerebral small vessel disease and that ALP was still significantly related to WMH volume after adjusting the renal function and excluding subjects with impaired kidney function (Ryu et al., 2014). ALP may play a role in the damage of WMHs to FOG in PD, but further research is needed.

Our study has some limitations: (1) This study is a single-center experiment with hospital-based study recruitment. (2) The visual rating system for WMHs proposed by Scheltens and colleagues is a semiquantitative method and some gaps between scores and the real impact of WMHs lesion may exist, although it is widely used and correlates well with volumetric measurements when WMHs are rated separately according to anatomical location. (3) We did not perform further analysis on the influence of WMHs on connectivity in the brain. DTI or other methods to explore regional connectivity are needed in the next step. (4) About the FOG, all patients in our study were evaluated during the OFF stage and other subtypes of FOG need to be further confirmed.

In conclusion, this study showed that WMHs play a role in PD patients with FOG. Notably, the burden of WMHs in the frontal regions exhibits a significantly correlated with FOG and potentially contributes to its occurrence and development. Additionally, the influence of ALP on FOG in PD patients may be through the augmentation of WMHs in the forehead.

## Data availability statement

The raw data supporting the conclusions of this article will be made available by the authors, without undue reservation.

## Ethics statement

The studies involving human participants were reviewed and approved by the Ethics Committee of the First Affiliated Hospital, Chongqing Medical University, China, in accordance with the Declaration of Helsinki. The patients/participants provided their written informed consent to participate in this study.

## Author contributions

XZ: conceptualization (equal), data curation (lead), writing—original draft preparation (lead), writing—review and editing (equal), and formal analysis (lead). ZD: data curation (equal), formal analysis (equal), and writing—original draft preparation (equal). XC: data curation (equal) and resources (equal). QY, HY, LY, JX, and XD: data curation (equal). HZ: data curation (equal) and formal analysis (equal). YH: resources (lead). DZ: resources (equal). OC: conceptualization (lead), funding acquisition, writing—original draft preparation (equal), and writing—review and editing (lead). JP: conceptualization (equal). All authors contributed to the article and approved the submitted version.

## Funding

This research was supported by the National Natural Science Foundation of China (nos. 81871002, 81471334, and 81100981), the National Key Clinical Specialties Construction Program of China, and Chongqing medical scientific research project (Joint project of Chongqing Health Commission and Science and Technology Bureau, 2022MSXM182).

## Conflict of interest

The authors declare that the research was conducted in the absence of any commercial or financial relationships that could be construed as a potential conflict of interest.

## Publisher’s note

All claims expressed in this article are solely those of the authors and do not necessarily represent those of their affiliated organizations, or those of the publisher, the editors and the reviewers. Any product that may be evaluated in this article, or claim that may be made by its manufacturer, is not guaranteed or endorsed by the publisher.

## Footnotes

^1^ www.statistical-modelling.de/lst.html
