# Supplementary figures and images for: White matter hyperintensities burden in the frontal regions is positively correlated to the freezing of gait in Parkinson’s disease
Source: Front Aging Neurosci. 2023 Apr 27;15:1156648. doi: 10.3389/fnagi.2023.1156648 (PMC10172504; doi:10.3389/fnagi.2023.1156648)

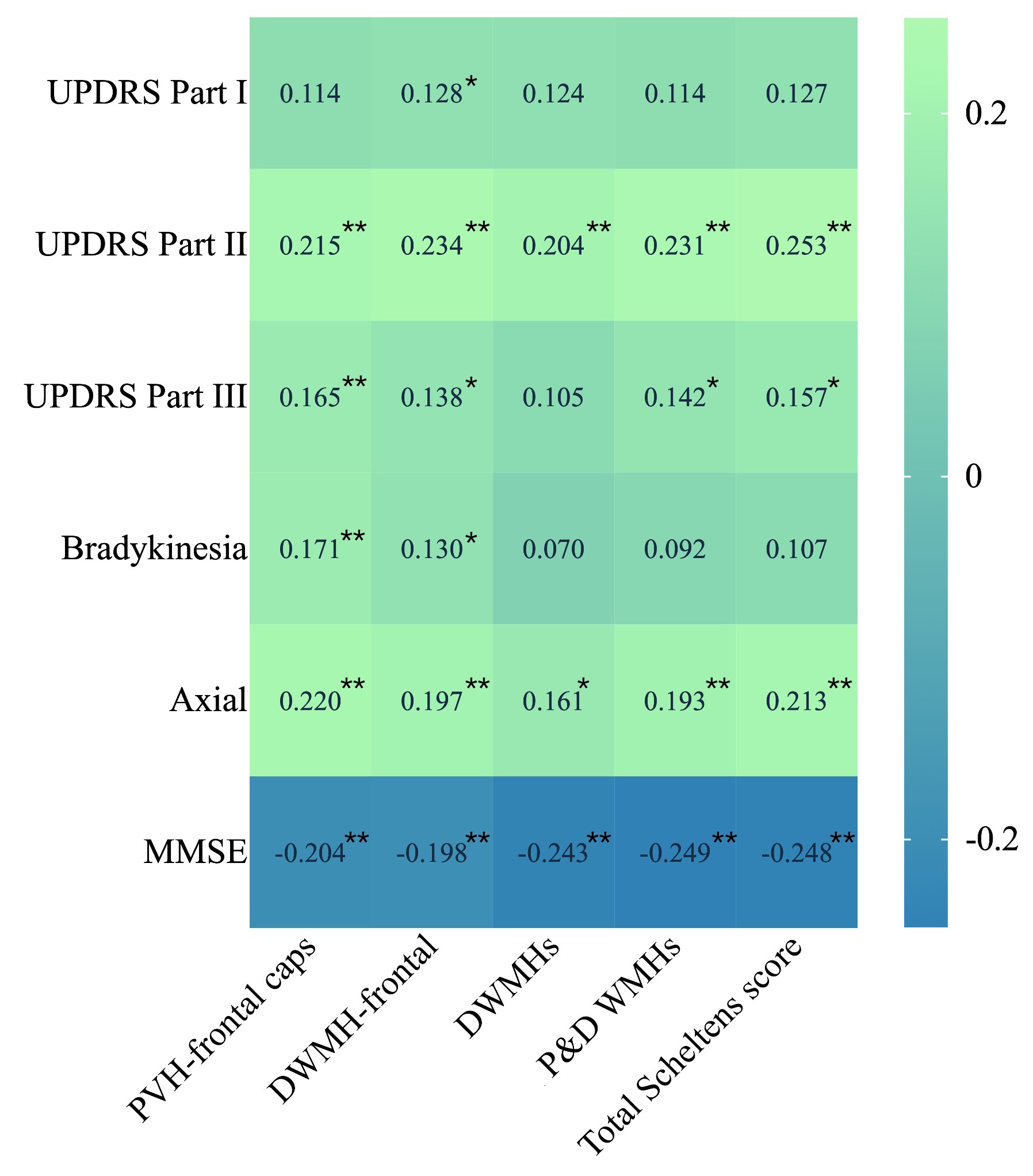

Supplement: Supplementary file 1 [file Image_1.JPEG]
