# Supplementary material for: White matter hyperintensities burden in the frontal regions is positively correlated to the freezing of gait in Parkinson’s disease
Source: Front Aging Neurosci. 2023 Apr 27;15:1156648. doi: 10.3389/fnagi.2023.1156648 (PMC10172504; doi:10.3389/fnagi.2023.1156648)
Supplement: Supplementary file 3 [file Table_1.docx]

**Supplementary Table 1**

**Binary Logistic regression analysis in five models showing the impact of WMHs on having FOG**

|  |  |  |  | 95% CI OR | |
| --- | --- | --- | --- | --- | --- |
| Variables | B | p value | OR | Lower limit | Upper limit |
| **Model 1. PVHs-frontal caps score** | |  |  |  |  |
| PVHs-frontal caps | 0.993 | 0.006^**^ | 2.699 | 1.337 | 5.450 |
| Sex | -0.378 | 0.327 | 0.685 | 0.322 | 1.458 |
| Disease duration | 0.126 | 0.032^*^ | 1.135 | 1.011 | 1.274 |
| L-dopa | 0.001 | 0.012^*^ | 1.001 | 1.000 | 1.002 |
| MDS-UPDRS Part I | -0.049 | 0.257 | 0.952 | 0.875 | 1.036 |
| MDS-UPDRS Part II | 0.107 | 0.020^*^ | 1.113 | 1.017 | 1.218 |
| MDS-UPDRS Part III | -0.109 | < 0.001^**^ | 0.897 | 0.853 | 0.943 |
| UPDRS bradykinesia | 0.118 | < 0.001^***^ | 1.125 | 1.072 | 1.180 |
| UPDRS axial | 0.528 | < 0.001^**^ | 1.696 | 1.325 | 2.171 |
| H&Y | -0.343 | 0.359 | 0.709 | 0.341 | 1.477 |
| HAMD | 0.034 | 0.443 | 1.035 | 0.948 | 1.129 |
| HAMA | -0.002 | 0.972 | 0.998 | 0.918 | 1.086 |
| **Model 2.** **DWMHs-frontal score** | |  |  |  |  |
| DWMHs-frontal | 0.233 | 0.009^**^ | 1.263 | 1.06 | 1.505 |
| Sex | -0.287 | 0.453 | 0.75 | 0.354 | 1.59 |
| Disease duration | 0.127 | 0.028^*^ | 1.136 | 1.014 | 1.272 |
| L-dopa | 0.001 | 0.032^*^ | 1.001 | 1.000 | 1.002 |
| MDS-UPDRS Part I | -0.056 | 0.186 | 0.945 | 0.87 | 1.027 |
| MDS-UPDRS Part II | 0.107 | 0.021^*^ | 1.113 | 1.017 | 1.218 |
| MDS-UPDRS Part III | -0.114 | < 0.001^**^ | 0.892 | 0.848 | 0.938 |
| UPDRS bradykinesia | 0.119 | < 0.001^**^ | 1.126 | 1.074 | 1.181 |
| UPDRS axial | 0.538 | < 0.001^**^ | 1.713 | 1.341 | 2.189 |
| H&Y | -0.193 | 0.6 | 0.825 | 0.401 | 1.695 |
| HAMD | 0.041 | 0.357 | 1.042 | 0.955 | 1.137 |
| HAMA | -0.001 | 0.986 | 0.999 | 0.919 | 1.087 |
| **Model 3. DWMHs score** |  |  |  |  |  |
| DWMHs | 0.090 | 0.047^*^ | 1.094 | 1.001 | 1.195 |
| Sex | -0.409 | 0.281 | 0.664 | 0.316 | 1.398 |
| Disease duration | 0.124 | 0.031^*^ | 1.132 | 1.011 | 1.266 |
| L-dopa | 0.001 | 0.028^*^ | 1.001 | 1.000 | 1.002 |
| MDS-UPDRS Part I | -0.059 | 0.170 | 0.943 | 0.867 | 1.025 |
| MDS-UPDRS Part II | 0.113 | 0.013^*^ | 1.120 | 1.024 | 1.224 |
| MDS-UPDRS Part III | -0.115 | < 0.001^**^ | 0.891 | 0.848 | 0.937 |
| UPDRS bradykinesia | 0.118 | < 0.001^**^ | 1.126 | 1.074 | 1.180 |
| UPDRS axial | 0.527 | < 0.001^**^ | 1.694 | 1.331 | 2.155 |
| H&Y | -0.192 | 0.597 | 0.825 | 0.406 | 1.680 |
| HAMD | 0.043 | 0.336 | 1.044 | 0.956 | 1.139 |
| HAMA | 0.001 | 0.984 | 1.001 | 0.921 | 1.087 |
| **Model 4. P&D WMHs score** | |  |  |  |  |
| P&D WMHs | 0.077 | 0.042^*^ | 1.08 | 1.003 | 1.164 |
| Sex | -0.409 | 0.282 | 0.664 | 0.316 | 1.399 |
| Disease duration | 0.122 | 0.032^*^ | 1.130 | 1.010 | 1.264 |
| L-dopa | 0.001 | 0.026^*^ | 1.001 | 1.000 | 1.002 |
| MDS-UPDRS Part I | -0.057 | 0.180 | 0.944 | 0.868 | 1.027 |
| MDS-UPDRS Part II | 0.112 | 0.014^**^ | 1.119 | 1.023 | 1.223 |
| MDS-UPDRS Part III | -0.115 | < 0.001^**^ | 0.891 | 0.848 | 0.937 |
| UPDRS bradykinesia | 0.119 | < 0.001^**^ | 1.126 | 1.074 | 1.180 |
| UPDRS axial | 0.528 | < 0.001^**^ | 1.696 | 1.332 | 2.159 |
| H&Y | -0.206 | 0.570 | 0.814 | 0.401 | 1.655 |
| HAMD | 0.042 | 0.350 | 1.043 | 0.955 | 1.138 |
| HAMA | 0.002 | 0.954 | 1.002 | 0.922 | 1.089 |
| **Model 5.** **Total Scheltens score** | |  |  |  |  |
| Total Scheltens score | 0.064 | 0.071 | 1.067 | 0.994 | 1.144 |
| Sex | -0.405 | 0.285 | 0.667 | 0.317 | 1.402 |
| Disease duration | 0.121 | 0.035^*^ | 1.128 | 1.009 | 1.262 |
| L-dopa | 0.001 | 0.024^*^ | 1.001 | 1.000 | 1.002 |
| MDS-UPDRS Part I | -0.056 | 0.190 | 0.946 | 0.870 | 1.028 |
| MDS-UPDRS Part II | 0.110 | 0.015^*^ | 1.116 | 1.021 | 1.220 |
| MDS-UPDRS Part III | -0.115 | < 0.001^**^ | 0.892 | 0.848 | 0.937 |
| UPDRS bradykinesia | 0.118 | < 0.001^**^ | 1.125 | 1.074 | 1.179 |
| UPDRS axial | 0.531 | < 0.001^**^ | 1.700 | 1.337 | 2.162 |
| H&Y | -0.200 | 0.578 | 0.818 | 0.404 | 1.660 |
| HAMD | 0.041 | 0.357 | 1.042 | 0.955 | 1.138 |
| HAMA | 0.003 | 0.942 | 1.003 | 0.923 | 1.090 |

OR: odds ratio; CI = confidence interval; WMHs: white matter hyperintensities; PVHs: periventricular hyperintensities; DWMHs: deep white matter hyperintensities; PVHs-frontal caps: the PVH in the anterior horns of the lateral ventricles; DWMHs-frontal: the DWMHs in the frontal lobe; P&D WMHs: the scores of PVHs and DWMHs; ^**^p< 0.01, ^*^p<0.05.
